# Long term visual outcomes in cataract surgery with bilateral implantation of the Extended Depth of Focus Intraocular Lens – Mini Well Ready type

**DOI:** 10.22336/rjo.2022.58

**Published:** 2022

**Authors:** Cristina Ariadna Nicula, Anca Maria Rednik, Ariadna Patricia Nicula, Adriana Elena Bulboacă, Dorin Nicula, Karin Ursula Horvath

**Affiliations:** * Department of Ophthalmology, “Iuliu Hațieganu” University of Medicine and Pharmacy, Cluj Napoca, Romania; ** Oculens Clinic, Cluj-Napoca, Romania; *** Department of Ophthalmology, County Emergency Hospital, Târgu Mureș, Romania; **** Department of Pathophysiology, „Iuliu Hațieganu” University of Medicine and Pharmacy, Cluj Napoca, Romania; ***** Department of Ophthalmology, “George Emil Palade” University of Medicine and Pharmacy Science and Technology, Târgu Mureș, Romania

**Keywords:** Mini Well Ready, EDOF-IOL, visual outcomes, patient satisfaction

## Abstract

**Purpose:** To evaluate the refractive results, visual outcomes and patients’ satisfaction of a new extended depth-of-focus (EDOF) intraocular lens (IOL).

**Setting:** Oculens Clinic, Cluj-Napoca, Romania.

**Design:** Retrospective, single-center, observational study.

**Methods:** 104 eyes of 52 patients undergoing cataract surgery with implantation of the Mini Well Ready EDOF-IOL (SIFI, Catania, Italy) were included in the study. Visual acuity at distance, intermediate and near was evaluated at 1, 6 and 12 months. Refractive results, contrast sensitivity, defocus curve and photic phenomena were also assessed.

**Results:** The mean age of patients was 66.04±7.82. A significant reduction of the spherical equivalent and improvement of corrected distance visual acuity (CDVA) was observed (p<0.0001) after the surgery. All eyes obtained a CDVA of 0.5 or better at one month. All eyes achieved a corrected intermediate visual acuity (CIVA) of 0.5 or better and a corrected near visual acuity (CNVA) of 0.5 or better. At 12 months the spherical equivalent was within -0.5- +0.5 diopters (D).

**Conclusions:** The EDOF-IOL offers good visual outcomes at distance, intermediate and near vision, providing an adequate contrast sensitivity and low rate of visual disturbances.

## Introduction

Cataract is one of the major causes of blindness worldwide. The purpose of cataract surgery is the restoration of the visual acuity by removing the opacified crystalline lens and implanting an artificial intraocular lens (IOL). During the past few years, cataract surgery became also a refractive procedure because of the advances of IOL technology [**1**] and the use of multifocal lenses increased by offering spectacles independence. The multifocal IOL technology (diffractive or refractive) allows patients to focus on images in several focal planes, obtaining spectacle independence and a large range of vision [**2**,**3**]. The major disadvantages of the multifocal IOLs are the increased photic phenomena and decreased contrast sensitivity [**4**]. Recently, the Extended Depth of Focus (EDOF) technology was introduced as a new concept of IOL. EDOF-IOL is a developing technology meant to enhance the range of vision, mainly at intermediate distance [**5**,**6**] avoiding undesirable photic phenomena and low contrast sensitivity (CS) [**7**]. Several studies showed good visual results with the first EDOF-IOL represented by the Tecnis Symfony (Johnson and Johnson Vision, USA), but the low contrast sensitivity and the presence of photic phenomena were the major undesired side effects, despite the excellent visual outcomes [**8**-**12**]. The Mini Well Ready (SIFI, Catania, Italy) is a new type of EDOF-IOL, with a double aspherical optic design with an addition of +3D based on positive and negative spherical aberrations in order to extend the range of focus and control the level of high order aberrations [**13**,**14**]. It is a biconvex hydrophilic-hydrophobic IOL with three different optical areas. The external area has a monofocal aspherical design, the intermediate area gives rise to a negative spherical aberration and the inner area produces a positive spherical aberration [**15**]. The passage between these three areas is gradual, to keep up a progressive vision. The overall diameter is 10.75 mm, the optic zone diameter is 6 mm, having an ultraviolet filter and fenestrated haptics with 5-degrees angulation.

Some studies reported excellent visual results at distance and intermediate vision, but limited at near vision, low rate of photic phenomena and increased depth of focus of the Mini Well Ready IOL [**15**-**18**]. Although many studies have been designed to show the efficacy and safety of EDOF-IOLs, especially regarding Symfony Technics EDOF- IOLs (Johnson and Johnson Vision, USA), there are only few studies that reported the vision quality, visual outcomes and photic phenomena of the Mini Well Ready EDOF-IOL [**5**,**10**,**15**,**16**,**19**,**20**].

The purpose of this study was to measure the visual outcome and refractive results at 1, 6 and 12 months, and patients’ satisfaction, in subjects who underwent cataract surgery and bilateral implantation of the EDOF-IOL Mini Well Ready. To our knowledge, there is no other study in the scientific literature so far reporting bilateral Mini Well Ready EDOF-IOL implantation in cataract surgery safety and efficacy in the Romanian population.

## Methods

A retrospective, observational study was conducted in Oculens Clinic, Cluj-Napoca, Romania. 104 eyes of 52 patients who underwent cataract surgery between April 2019 and January 2020 with bilateral implantation of the EDOF-IOL Mini Well Ready (SIFI, Catania, Italy) were included in the study. The present study adhered to the tenets of the declaration of Helsinki and was approved by the ethical committee of Oculens Clinic (approval 2/ 2022).

Inclusion criteria were: male and female patients older than 65 years, diagnosed with different types and stages of age-related cataract and uncomplicated surgery.

Exclusion criteria were: any corneal pathology (scars, Fuchs disease, keratoconus, pellucid marginal dystrophy), astigmatism higher than 1.0 D, previous eye surgery, pseudoexfoliation, pupil abnormalities (non-reactive, extremely small pupils under photopic conditions), very shallow anterior chamber, proliferative diabetic retinopathy, medically uncontrolled glaucoma, severe optic nerve atrophy, microphthalmos, amblyopia, recurrent inflammation of anterior and/ or posterior segment (chronic uveitis), monocular patients, severe dry eye, patients with too high or unrealistic expectations.

Before surgery, patients underwent a complete ocular exam including the pre-operative uncorrected distance visual acuity (UDVA) and best-corrected distance visual acuity (CDVA), refractometry, keratometry (Kmax, Kmin) using the autorefractometer (Topcon auto kerato-refractometer, KR 8900, Japan), slit lamp examination of the anterior segment and eye fundus (Slit-Lamp BX 900, Haag-Streit AG), intra-ocular pressure (Haag-Streit AT 900 aplanotonometer), endothelial cell count (Konan SP-9000, Hyogo, Japan). Tear film stability was checked by measuring the tear break-up time (TBUT) and Schirmer test. Optical biometry by interferometry was performed using the IOL Master 700 (Carl Zeiss Meditec AG, Germany) to establish the axial length and dioptric power of the IOL, using the fourth-generation formula Barrett Universal II (Holladay IOL consultant, Houston, TX, USA and www.apacrs.org/barrett_universal2). In every case we also used the immersion ultrasound biometry (OcuScan, R and P Ophthalmic Ultrasound System, Alcon). The used A constant was 118.6. The used formulas were concordant with the axial length of the eye: Haigis or Hoffer Q for axial length (AXL) < 22 mm, SRK T, Holliday or Hoffer Q for AXL between 22-24.5 mm, Holladay for AXL between 24.5-26 and SRK T for AXL ˃ 26 mm [**21**]. The targeted postoperative refraction was emmetropia in every case. An important issue was to measure total astigmatism, high order aberrations (HOAs), spherical aberration and corneal asphericity using the Pentacam® topographer (HR Premium; Oculus Optikgerate GmbH, Wetzlar, Germany), angle kappa and pupil size in photopic and scotopic conditions [**21**]. After the ocular exam, a discussion with the patient regarding possible intraoperative and postoperative complications and the advantages (spectacle independence, better vision at distance and intermediate, low rate of haloes) and disadvantages (necessity of wearing glasses at certain activities, suboptimal near vision) of the EDOF-IOL took place. An informed written consent was signed by all patients before surgery, in which they agreed with the cataract surgery and Mini Well Ready EDOF-IOL implantation.

Cataract surgery was performed by phacoemulsification (Centurion, Alcon, Fort Worth, TX, USA) under topical anesthesia, through a corneal suture less 2.2 mm incision performed on the most astigmatic meridian, manually consistent curvilinear capsulorhexis, hydro dissection and hydrodelineation, quick chop technique for phacoemulsification, irrigation-aspiration of the cortex, posterior capsule polishing and in the bag implantation of the preloaded Mini Well Ready EDOF-IOL. Finally, the centration of the IOL was done using the Purkinje images [**22**,**23**]. Cataract surgery of the opposite eye was performed after 3 days to 6 weeks after the first operated eye, concordant with the lens opacification grade and patient’s desire. Postoperatively, the patients followed a topical treatment with Tobradex (Alcon, Fort Worth, TX, USA) 5 times per day, for 1 month. The follow-up of the patients was at 24 hours after surgery, 1, 6 and 12 months. At each visit, UDVA, best-corrected distance visual acuity (CDVA), uncorrected intermediate visual acuity (UIVA), best-corrected intermediate visual acuity (CIVA), uncorrected near visual acuity (UNVA: 40 cm) and best-corrected near visual acuity (CNVA: 40 cm), monocularly and binocularly were performed. For scientific purposes, we transformed the decimal values of VA into Logarithm of Minimum Angle of resolution (logMar scale). The manifest refraction was performed using the maximum plus refraction technique (“push plus”) [**24**]. Early Treatment Diabetic Retinopathy Study (ETDRS) charts and additional lenses from +1D to -4D and with 0.5D additive values were used in order to achieve the binocular best distance-corrected defocus curve [**25**,**26**]. The binocular contrast sensitivity test (CS) was assessed in photopic and mesopic conditions using the Pelly-Robson contrast chart. On the last clinical follow-up visit, a questionnaire was completed by all the patients in order to establish the subjective satisfaction with the outcome and the presence of glare or haze. The questionnaire was conceived by our clinic for all the patients who received multifocal lenses (other types as well) and included the following questions:

1. Do postoperative halos affect you?

2. Is your visual acuity in dim light condition satisfactory after the surgery?

3. Is your visual acuity at a distance, intermediate and near distance satisfactory after the surgery?

4. How satisfied are you with your spectacles’ independence? (1-very satisfied, 2-satisfied, 3-thankful, 4-unhappy, 5-very unhappy)

## Statistics

Data were recorded as mean ± standard deviation (± SD). Results at different follow-up visits were analyzed with the paired t-test. P values < 0.001 were considered as high statistically significant. The software used for the statistical analysis was Graph-pad/ v.5 Graph-pad holding.

## Results

104 eyes of 52 patients, diagnosed with age-related cataract, underwent cataract surgery with intrabag implantation of the EDOF-IOL Mini Well Ready. The mean age was 66.04 ± 7.82 years old (range 50-87; males:females = 1:1). The power of the implanted EDOF-IOL ranged from 8 to 37 D (mean: 20.13 ± 4.84 D). Preoperatively, the mean corneal asphericity (Q value at 8 mm) was -0.28 ± 0.11. The preoperative parameters are presented in **Table 1**.

**Table 1:** Preoperative parameters

	Mean (SD)	Min	Max
Kmin (mm)	42.92±1.36	40	46
Kmax (mm)	43.58±1.44	40.5	46.6
DeltaK	-0.66±0.35	-2	0
Axial length (mm)	23.81±1.56	19.48	28.74
ACD (mm)	3.17±0.47	2.12	4.53
Dioptric IOL power (D)	20.13±4.84	8	37
Preoperative CDVA (logMar)	0.55±0.13	1	0.22
Photopic pupil diameter	3.04±0.03	2.99	3.1
Scotopic pupil diameter	4.16±0.03	4.1	4.19
Q value	-0.28±0.11	-0.55	0.1
Abbreviations: Kmin = minimum keratometry; Kmax = maximum keratometry; ACD = anterior chamber depth; CDVA = best-corrected distance visual acuity			

*Refractive results*

The mean predicted refraction was 0.14 ± 0.13 D. The mean accomplished refraction was 0.37 ± 0.53 D at 4 weeks. Almost 39.05% of the patients were between -0.25 and +0.25D of the target refraction, with 66.67% eyes between -0.5 and +0.5D of planned correction.

At 1 month, the mean postoperative SE was -0.37 ± 0.53D. 60.95% of the eyes were between -0.5D and +0.5D, 39.05% eyes between -1 and +1D. At 6 months postoperative, 66.35% of the eyes were between -0.5 and +0.5 D. At 12 months after cataract surgery, 89.9% of the eyes had a stable manifest refraction (p > 0.05). The mean postoperative cylinder was -0.57 ± 0.31 D (range -1.5 to 0 D). What should be mentioned is that the auto-keratometry refraction is not always the true one and the induced astigmatism must be evaluated by postoperative keratometry.

*Visual outcomes*

The monocular visual outcomes during the follow-up period are summarized in **Table 2**. A significant statistical improvement was noted regarding postoperative monocular logMar UDVA, UIVA, UNVA, CDVA, CIVA, CNVA in comparison with preoperative visual acuities (p < 0.0001), with no significant difference between 6 and 12 months after cataract surgery (p > 0.05).

The binocular visual outcome at all visits is reported in **Table 3**. Binocular values of log Mar CDVA, CIVA, CNVA were statistically significantly improved (p < 0.0001) compared with the preoperative values of the visual acuity.

**Table 2:** Postoperative monocular visual outcome

		Post-operative			Preoperative p value vs	Preoperative p value vs	Preoperative p value vs	6 months p value vs
	Preoperative	4 weeks	6 months	1 year	4 weeks postoperative	6 months postoperative	1 year postoperative	1 year postoperative
UDVA	0.7±0.11	0.09±0.13	0.09±0.13	0.09±0.14	<0.0001	<0.0001	<0.0001	0.8218
UIVA	0.64±0.15	0.19±0.24	0.19±0.17	0.19±0.18	<0.0001	<0.0001	<0.0001	0.9369
UNVA	0.6±0.13	0.23±0.23	0.23±0.19	0.23±0.20	<0.0001	<0.0001	<0.0001	0.9718
CDVA	0.55±0.13	0.04±0.12	0.04±0.11	0.04±0.14	<0.0001	<0.0001	<0.0001	0.7107
CIVA	0.5±0.16	0.13±0.11	0.13±0.19	0.13±0.20	<0.0001	<0.0001	<0.0001	0.9412
CNVA	0.48±0.16	0.17±0.13	0.17±0.21	0.17±0.22	<0.0001	<0.0001	<0.0001	0.9742
Abbreviations: UDVA = uncorrected distance visual acuity, CDVA = best-corrected distance visual acuity; UIVA = uncorrected intermediate visual acuity, CIVA = best-corrected intermediate visual acuity; UNVA = uncorrected near visual acuity, CNVA = best-corrected near visual acuity								

**Table 3:** Postoperative binocular visual outcomes

		Post-operative			Preoperative p value vs	Preoperative p value vs	Preoperative p value vs	6 months p value vs
	Preoperative	4 weeks	6 months	1 year	4 weeks postoperative	6 months postoperative	1 year postoperative	1 year postoperative
CDVA	0.50±0.13	0.03±0.09	0.03±0.10	0.03±0.13	<0.0001	<0.0001	<0.0001	0.9447
CIVA	0.47±0.16	0.13±0.09	0.13±0.19	0.13±0.20	<0.0001	<0.0001	<0.0001	0.9715
CNVA	0.46±0.18	0.17±0.13	0.17±0.21	0.17±0.22	<0.0001	<0.0001	<0.0001	0.9750
Abbreviations: CDVA = best-corrected distance visual acuity; CIVA = best-corrected intermediate visual acuity; CNVA = best-corrected near visual acuity								

Postoperatively, the mean UDVA and CDVA were 0.7 ± 0.11 logMAR and 0.55 ± 0.13 logMAR, respectively. The mean UIVA (at 80 cm) and CIVA were 0.64 ± 0.15 logMAR and 0.5 ± 0.16, respectively. The mean UNVA (at 40 cm) and CNVA was 0.16 ± 0.13 logMAR and 0.48 ± 0.16 logMAR, respectively, with a mean minimum add of +1.50 D. Although, the UNVA and CNVA decreased below 0.3 logMAR, the achieved vision was a functional one, demanding a low correction for certain activities.

**Fig. 1:**
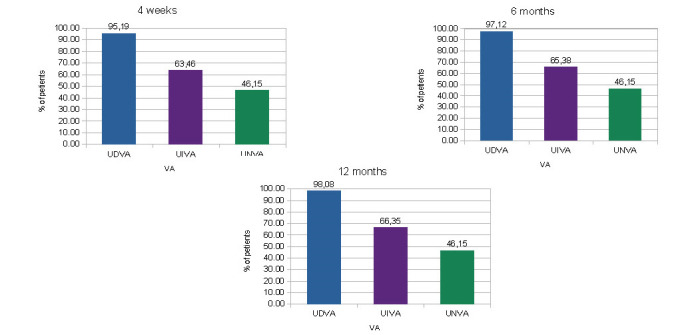
Distribution of monocular postoperative UDVA, UIVA and UNVA of 0.2 logMar or better

**Fig. 2:**
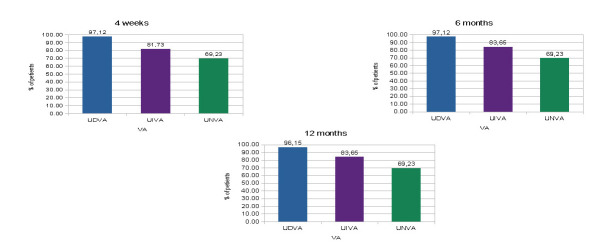
Distribution of binocular postoperative UDVA, UIVA and UNVA of 0.2 logMar or better

As it appeared, a total of 95.19%, 63.46% and 46.15% eyes obtained a monocular UDVA, UIVA and UNVA of 0.2 logMar or better, respectively, at 4 weeks, 97.12%, 65.38% and 46.15% eyes achieved a monocular UDVA, UIVA and UNVA of 0.2 logMar or better, respectively, at 6 months and 98.08%, 66.35% and 46.15% eyes achieved a monocular UDVA, UIVA and UNVA of 0.2 logMar or better, respectively, at 1 year (**Fig. 1**). A total of 97,12%, 81.73% and 69.23% eyes obtained a binocular UDVA, UIVA and UNVA of 0.2 logMar or better, respectively, at 4 weeks, 97.12%, 83.65% and 69.23% eyes achieved a binocular UDVA, UIVA and UNVA of 0.2 logMar or better, respectively, at 6 months and 96.15%, 83.65% and 69.23% eyes achieved a binocular UDVA, UIVA and UNVA of 0.2 logMar or better, respectively, at 1 year (**Fig. 2**).

**Fig. 3:**
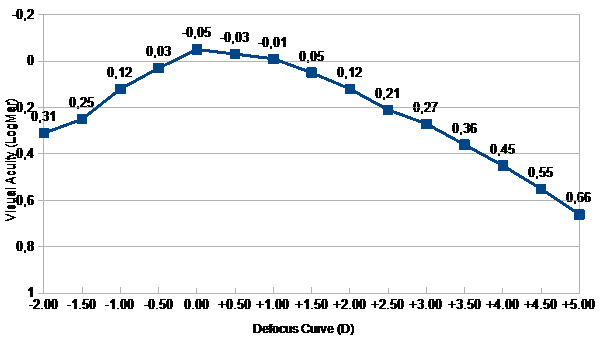
Defocus curve

The defocus curve declined increasingly as the reading distance reduced and showed optimal visual acuity from +1.00 to −2.00 D (40 cm). At a visual acuity of 0.27 logMar, Mini Well Ready provided a range of +3 D and at 0.05 logMar offered a range of +1.5 D (**Fig. 3**). The Mini Well binocular defocus curve showed that visual acuity of 0.3 logMAR or better was maintained from 0.0 D to almost -2D, certifying the extended depth of focus of this intraocular lens.

*Contrast sensitivity*

**Fig. 4:**
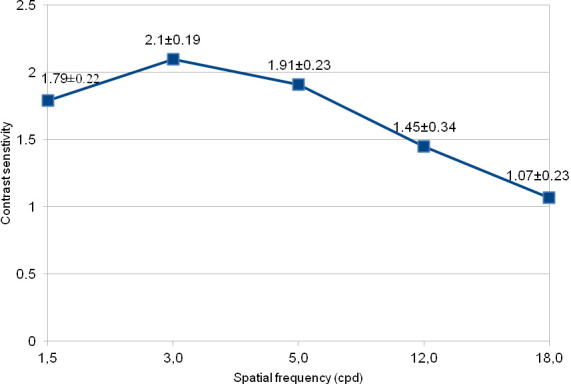
Monocular contrast sensitivity

The mean values of CS (expressed as log10 CS) are presented in **Fig. 4**. The values were 1.79 ± 0.22 at 1.5 cycle per degree (cpd), 2.1 ± 0.19 at 3 cpd, 1.91 ± 0.23 at 5 cpd, 1.45 ± 0.34 at 12 cpd and 1.07 ± 0.23 at 18 cpd. Mini Well Ready has an adequate photopic CS, within normal values.

*Complications*

None of the patients developed intraoperative complications. One patient complained about halos and another patient about glare (2 patients overall; 2.94%).

*Patient satisfaction*

At the last follow-up visit, according to the questionnaires, 85.6% of the patients reported a high satisfaction (=1-very satisfied) with the outcomes after surgery and bilateral implantation of Mini Well EDOF-IOL.

## Discussion

In the past few years, IOL multifocal technology encountered an excellent progress in cataract surgery regarding the patient’s visual outcomes for distance, intermediate and near. The multifocal IOL should be chosen considering the patient’s daily activities, work, and the need to see well at a certain distance. The new EDOF-IOL uses a technology that can deliver a continuum focus by introducing the spherical aberration and tries to offer better contrast sensitivity, low photic phenomena and a good visual acuity at distance and intermediate in comparison with the multifocal IOLs. Most of the performed studies took into consideration the binocular uncorrected visual outcomes of the Symfony IOL (Johnson and Johnson Vision) and comparative results between EDOF-IOLs and multifocal or monofocal IOLs. **Table 4** summarizes the data from the studies mentioned previously.

**Table 4:** Binocular uncorrected visual outcomes in different studies

Publication	Follow-up Period (months)	Surgery	IOL	Number of eyes	UDVA (logMar) binocular	UIVA (logMar) binocular	UNVA (logMar) binocular
Cochener et al. [**7**], 2018	6	Cat	EDOF_Simfony/ Trifocal Panoptix/ Fine Vision	40/ 40/ 40	0.01±0.03 0.03±0.05 0.02±0.06	0.3±0.14 0.36±0.16 0.42±0.09	0.32±0.13 0.19±0.04
Savini et al. [**15**], 2019	1-2	Cat	EDOF_MiniWell	164	0.11±0.07	0.14±0.69	0.38±0.15
Savini et al. [**16**], 2018	1	Cat/ CLE	EDOF_MiniWell/Multifocal Restore	35/ 39	0.04±0.6 0.03±0.06		0.26±0.13
Pilger [**19**], 2018	3	Cat	EDOF_Simfony/MonofocalTechnisZCB00	30/ 30	-0.02±0.08 0.03±0.11	-0.13±0.07 0.00±0.06	0.11±0.07 0.26±0.1
Auffarth et al. [**20**], 2020	2-4	Cat	EDOF_MiniWell	68	-0.01±0.15	0.03±0.10	0.10±0.11
Monaco et al. [**27**], 2017	4	Cat/ RLE	EDOF_Simfony/Panoptix/SN60 WF	40/ 40/ 40	NA	NA	NA
Escandon-Garcia et al. [**28**], 2018	3	Cat	EDOF_Simfony/Trifocal-Panoptix; FineVision	30/ 14/ 46	0.08±0.01 0.08±0.09	NA	NA
Ruiz-Mesa et al. [**29**], 2018	29	Cat	EDOF_Simfony/Trifocal Panoptix;AT Lisa	40/40/40	0.05±0.12 0.0±0.03	NA	NA
Mencucci [**30**], 2018	4	Cat	EDOF_Simfony/Trifocal-Panoptix; FineVision	30/ 14/ 46	-0.04±0.05 -0.01±0.06	0.07±0.07 0.14±0.69	0.25±0.08 0.17±0.05
Pedrotti [**31**], 2020	3	Cat	EDOF_MiniWell/Monofocal Mini Ready	50/ 50	0.01±0.10 0.11±0.16	0.06±0.10 0.26±0.15	0.10±0.09 0.49±0.17
Kretz FT et al. [**32**], 2015	12	Cat	EDOF_Simfony/Trifocal Fine Vision	40/ 40	0.01±0.02 0.01±0.03	0.09±0.08 0.11±0.08	0.17±0.06 0.06±0.07
Abbreviations: UDVA = uncorrected distance visual acuity; UIVA = uncorrected intermediate visual acuity; UNVA = uncorrected near visual acuity							

In our study, 98.08% and 66.35% of the patients achieved an UDVA and UIVA of 0.2 or better logMar monocularly at 1 year, respectively, and 46.15% of the patients achieved a UNVA of 0.2 logMar at 1 year. In binocularity, 96.15%, 83.65% and 69.23% achieved a UDVA, UIVA, UNVA respectively, at 1 year. Similar results were revealed by Savini et al. [**15**] in a prospective study including 97 patients who underwent cataract surgery with Mini Well EDOF-IOL implantation and achieved a binocular CDVA value of 0.0 ± 0.5 logMar. Moreover, the same author demonstrated that the Mini Well Ready EDOF-IOL achieved alike UDVA vision to previous multifocal lenses. In a retrospective comparative study, Savini et al. [**16**] revealed that there was no statistically significant difference upon the CDVA between the EDOF-IOLs and Restore SV25T multifocal lenses (Alcon, Texas, SUA). Furthermore, the distance visual outcomes were similar with those reported by Jonker et al. [**33**] in a prospective randomized clinical trial, which compared the visual results in patients with cataract surgery and bilateral implantation of Fine vision Micro F trifocal IOLs (Physiol SA, Liège, Belgium) or Acrysof IQ Restore +3D bifocal IOLs (Alcon Laboratories, Inc., Fort Worth, Tx, USA).

In our study, the best UIVA was achieved at 80 cm. Giers et al. [**13**] showed that the patients with EDOF-IOL preferred an intermediate reading distance of 62.8 cm. In a comparative study, Cochener et al. [**7**] showed no statistically significant difference of UIVA between the trifocal intraocular lenses and Symfony EDOF lenses. Furthermore, Savini et al. [**15**] reported very good outcomes regarding the UNVA, with a significant improvement in bilateral Mini Well EDOF-IOLs implantation.

Our findings showed that at a visual acuity of 0.27 logMar, Mini Well Ready provided a range of 3 D and at 0.05 logMar offered a range of 1.5 D. Similar results were demonstrated by Savini et al. [**16**], who noted that the increased depth of focus was provided through 2.0 D defocus and the greatest performance was achieved at -1.0 and -1.5 D. Furthermore, Dominguez-Vincent et al. [**17**] revealed that even though the lens displayed excellent optical quality at a large defocus range, the visual outcomes are dependent of the pupil size.

Regarding CS, our findings revealed that the Mini Well EDOF-IOL can offer a proper CS, being within normal values. Similar results were demonstrated by Savini et al. [**15**], who concluded that both Mini Well EDOF-IOL and Acrysof ReStore SV25T0 (Alcon Laboratories, Inc., Fort Worth, Tx, US) have similar outcomes in terms of CS. Mencucci et al. [**30**] revealed that the CS with Symfony EDOF-IOLs was statistically significantly better in comparison with the CS with Panoptix (Alcon Laboratories, Inc., Fort Worth, Tx, USA) and the one with AT Lisa Tri 839 MP (Carl Zeiss Meditec AG).

In the present study, glare and halos were present only in 2.94% of the cases. The low incidence of photic phenomena with the Mini Well EDOF IOLs in comparison with the multifocal IOLs [**15**,**28**,**32**] can be explained by the absence of the concentric diffractive rings on the optics of the IOL. Moreover, the central area that induces a positive spherical aberration has a smaller size (3 mm) in comparison with the diffractive area of the multifocal IOL (3.4 mm).

In a European multicentric, prospective study, Auffarth et al. [**20**], Pilger et al. [**19**] and Pedrotti et al. [**31**] reported no significant difference in halos between EDOF and monofocal IOLs [**19**,**31**]. Furthermore, the FDA Clinical Trial revealed that halos were more frequently present in EDOF-IOLs comparative with the monofocal IOLs [**34**]. Several studies reported no statistically significant difference in photic phenomena between trifocal and EDOF-IOLs [**7**,**11**,**27**,**29**,**30**]. Savini et al. [**15**] reported the presence of halos in 5% of Mini Well EDOF-IOLs and in 27% of multifocal IOLs, noting that the mean intensity and size of halos were statistically significant lower in the Mini Well EDOF-IOL group in comparison with the multifocal IOLs.

In our study, 85.6% of the patients were satisfied with the visual outcomes. Similar results were revealed by Auffarth et al. [**20**] who showed that 85.29% of the patients were perfectly content with the visual results of Mini Well EDOF-IOL. 

More studies are still required to confirm the efficiency and safety of Mini Well EDOF-IOL.

This paper has several limitations that should be acknowledged. This was a retrospective study and there was no randomization, no comparison with other multifocal IOLs. The satisfaction of the patients was not established with a specific and validated questionnaire. However, the strength of our paper resides in the relatively large sample size compared to previous reports and the long-term follow-up of these patients, adding to previous knowledge on this subject. To our knowledge this is the first study conducted in Romania.

## Conclusion

The Mini Well EDOF-IOL offers good visual outcomes at distance, intermediate and near distances, providing an adequate contrast sensitivity and low rate of visual disturbances.

**Value statement**

**What was known**

• There are only few studies up to present that report visual outcomes, quality of vision and photic phenomena of the Mini Well Ready EDOF-IOL.

• Previous studies reported excellent visual results at distance and intermediate vision, but limited at near vision, low rate of photic phenomena and increased depth of focus of the Mini Well Ready IOL.

**What this paper adds**

• This is the first published experience reporting bilateral Mini Well Ready EDOF-IOL implantation in cataract surgery in the Romanian population.

• This single-center observational study confirms that the Mini Well EDOF-IOL offers good visual outcomes at distance, intermediate and near distances, providing an adequate contrast sensitivity and low rate of visual disturbances.

**Conflict of Interest statement**

All authors declare that they have no conflicts of interest. 

**Informed Consent and Human and Animal Rights statement**

All appropriate patient consent forms for the publication of case details and images have been obtained from all individuals included in this study.

**Authorization for the use of human subjects**

Ethical approval: The research related to human use complies with all the relevant national regulations, institutional policies, is in accordance with the tenets of the Helsinki Declaration, and has been approved by the ethical committee of Oculens Clinic, Romania (approval 2/ 2022).

**Acknowledgements**

Editorial assistance in the preparation of this article was provided by Laura C Collada Ali (Medical Writing Consultant). Support for this assistance was funded by SIFI S.p.A., Italy. The investigators thank the patients who were object of this study for their involvement.

**Sources of Funding**

The present study has been funded and sponsored by SIFI S.p.A (Italy).

**Disclosures**

The authors declare that they have no competing interests in the paper. The authors have no potential conflicts of interest with respect to the research, authorship, and/ or publication of this article.

**Authorship**

All authors adhere to the following guidelines for authorship: ICMJE, Defining the Role of Authors and Contributors, Transparency in authors’ contributions and responsibilities to promote integrity in scientific publication, McNutt at al., PNAS February 27, 2018.
